# Incidence trends and survival prediction of urothelial cancer of the bladder: a population-based study

**DOI:** 10.1186/s12957-021-02327-x

**Published:** 2021-07-26

**Authors:** Hairong He, Tianjie Liu, Didi Han, Chengzhuo Li, Fengshuo Xu, Jun Lyu, Ye Gao

**Affiliations:** 1 Clinical Research Center, The First Affiliated Hospital of Xi’an Jiaotong University, Xi’an, Shaanxi People’s Republic of China; 2 School of Public Health, Xi’an Jiaotong University Health Science Center, Xi’an, Shaanxi People’s Republic of China; 3 Department of Urology, The First Affiliated Hospital of Xi’an Jiaotong University, Xi’an, Shaanxi People’s Republic of China; 4 Department of Clinical Research, The First Affiliated Hospital of Jinan University, Guangzhou, People’s Republic of China; 5 Department of Emergency, The First Affiliated Hospital of Xi’an Jiaotong University, 277 West Yanta Road, Xi’an, Shaanxi 710061 People’s Republic of China

**Keywords:** Urothelial cancer of the bladder, Incidence, Survival, Nomogram

## Abstract

**Background:**

The aim of this study is to determine the incidence trends of urothelial cancer of the bladder (UCB) and to develop a nomogram for predicting the cancer-specific survival (CSS) of postsurgery UCB at a population-based level based on the SEER database.

**Methods:**

The age-adjusted incidence of UCB diagnosed from 1975 to 2016 was extracted, and its annual percentage change was calculated and joinpoint regression analysis was performed. A nomogram was constructed for predicting the CSS in individual cases based on independent predictors. The predictive performance of the nomogram was evaluated using the consistency index (C-index), net reclassification index (NRI), integrated discrimination improvement (IDI), a calibration plot and the receiver operating characteristics (ROC) curve.

**Results:**

The incidence of UCB showed a trend of first increasing and then decreasing from 1975 to 2016. However, the overall incidence increased over that time period. The age at diagnosis, ethnic group, insurance status, marital status, differentiated grade, AJCC stage, regional lymph nodes removed status, chemotherapy status, and tumor size were independent prognostic factors for postsurgery UCB. The nomogram constructed based on these independent factors performed well, with a C-index of 0.823 and a close fit to the calibration curve. Its prediction ability for CSS of postsurgery UCB is better than that of the existing AJCC system, with NRI and IDI values greater than 0 and ROC curves exhibiting good performance for 3, 5, and 8 years of follow-up.

**Conclusions:**

The nomogram constructed in this study might be suitable for clinical use in improving the clinical predictive accuracy of the long-term survival for postsurgery UCB.

## Introduction

Urothelial cancer of the bladder (UCB) is the most common pathological type of bladder cancer, and its incidence is especially high in Western countries [1, 2]. The incidence of this cancer is closely related to tobacco consumption and exposure to occupational carcinogens [3, 4]. However, the incidence of UCB may have changed over the past few decades due to industrial developments, the implementation of policies for controlling tobacco, and progress in disease diagnosis and treatment [5, 6]. There have been few analyses of the incidence of UCB despite many studies researching the incidence trends of bladder cancer [7].

UCB is most frequently diagnosed in males and people older than 55 years [7]. Surgical resection is the mainstay treatment for UCB, but many people—especially those presenting with muscle invasion—have poor outcomes despite receiving surgery and systemic treatment [8]. Although prognostic factors for UCB have been reported [9–16], reliable nomograms for individualized predictions of the long-term survival of postsurgery patients with UCB are still lacking.

Given the aforementioned situation, this study analyzed trends in the incidence of UCB and established a nomogram based on a Cox proportional-hazards regression analysis of the prognostic factors for predicting the survival of UCB after surgery based on data obtained from the Surveillance, Epidemiology, and End Results (SEER) database [17].

## Methods

### Data collection and definition

The data were extracted retrospectively from the SEER database and downloaded using SEER*Stat software (version 8.3.6, National Cancer Institute). To identify UCB patients, we searched the database using the tumor-site ICD-9 codes (C67.0–C67.9) and ICD-O-3 code (8130/3). To analyze the trends in the incidence of UCB, the age-adjusted incidence rate of UCB diagnosed from 1975 to 2016 was calculated.

To establish a nomogram for analyzing survival, the following variables for UCB were extracted from the SEER database: age at diagnosis, sex, ethnic group, primary site, grade, metastasis stage, derived AJCC stage, regional lymph nodes removed, radiation status, chemotherapy status, insurance status, marital status, tumor size, survival time, and cancer-specific death status. We only included patients who received surgery, which were identified with “Surgery performed” record on the item “Reason no cancer-directed surgery.” Other exclusion criteria were (1) only autopsy findings being available, (2) diagnosis based on direct visualization without microscopic confirmation, (3) not the first malignant primary indicator, and (4) incomplete information for the above-listed variables.

### Statistical analyses

The data for the age-adjusted incidence rate of UCB from 1975 to 2016 was used to calculate the annual percentage change (APC) in the incidence using the weighted least-squares method. Joinpoint regression analysis (version 4.7.0, Joinpoint, IMS, Calverton, MD, USA) was performed to delineate trends in the incidence of UCB from 1975 to 2016. Considering the large difference in the incidence between males and females, the APC analysis and the joinpoint regression analysis were performed with stratification by sex.

All of the patients included in the cancer-specific survival (CSS) analysis were randomly divided into a training cohort and a validation cohort at the ratio of 7:3. We first used the data in the training set to find independent prognostic factors and construct a nomogram, and then applied the data to the validation cohort to evaluate the distinguishability, calibration, and clinical effectiveness of the prediction model.

Differences in the distribution of categorical variables between the training cohort and validation cohort were estimated using the chi-square test. Differences in age between the two cohorts were assessed using Student’s *t* test, and differences in survival time were assessed using the log-rank test. Statistical analyses to identify risk factors were performed by applying the backward stepwise selection method of multivariable Cox regression to the training cohort. A nomogram was then established based on the identified risk factors.

The distinguishability of the nomogram was evaluated using the consistency index (C-index) calculated by Harrell’s C statistic, the net reclassification index (NRI), and the integrated discrimination improvement (IDI). The C-index was used to describe the difference between the real values and those predicted by the model. This index ranges from 0.5 (no discrimination) to 1 (excellent discrimination), with a value of ≥ 0.70 indicating that the distinguishability of the prediction model is acceptable. Values of NRI and IDI of > 0 (compared with the traditional AJCC staging system) indicate that the prediction ability of the nomogram is better than that of the AJCC staging system, while negative values would indicate that it is inferior.

The calibration of the nomogram was evaluated using a calibration plot, on which the abscissa shows the predicted values for different groups and the ordinate shows the actual probabilities. The value points for different groups are connected by line segments to form a calibration line. A calibration curve that is closer to the standard line of *y* = *x* indicates a smaller error between the model’s prediction and the actual situation, and hence a better calibration capability of the model. The clinical effectiveness of the nomogram was evaluated using the receiver operating characteristics (ROC) curve.

Statistical analyses were performed using the R software (version 3.5.1; https://www.r-project.org/). Statistical significance was defined as a two-sided probability value of < 0.05.

## Results

### Incidence trends

**Fig. 1:**
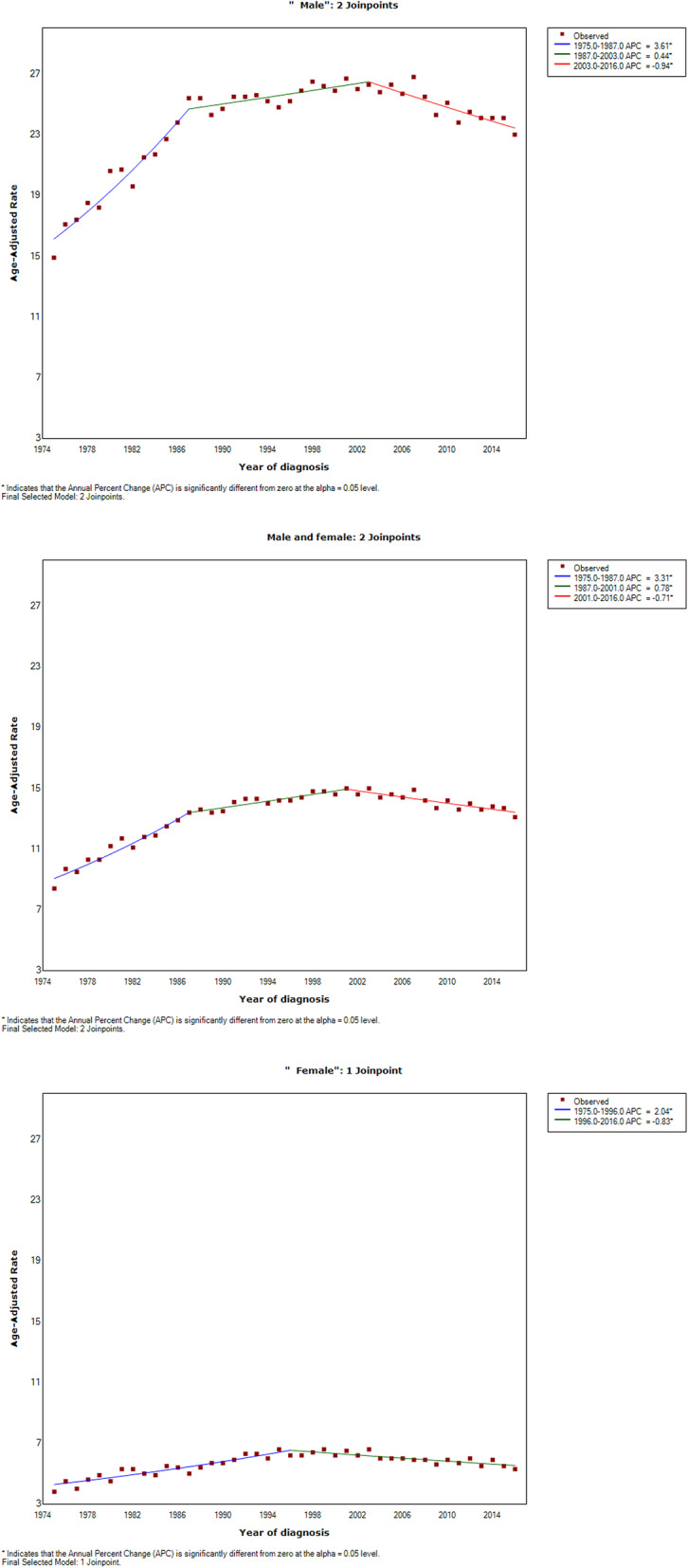
The incidence trends by sex of urothelial cancer of the bladder from 1975 to 2016

The age-adjusted incidence rate of UCB increased from 8.4 per 100,000 persons in 1975 to 13.1 per 100,000 persons in 2016. The joinpoint regression analysis revealed there were two join points (Fig. 1). The incidence rate showed a rapidly increasing trend from 1975 to 1987, with an APC of 3.3% (95% confidence interval [CI] = 2.8–3.9%, *P* < 0.0001), while this increase slowed from 1988 to 2001 (APC = 0.8%, 95% CI = 0.4–1.1%, *P* < 0.0001), and was followed by a decreasing trend from 2002 to 2016 (APC= −0.7%, 95% CI= −0.9% to −0.5%, *P* < 0.0001).

Among male UCB patients, the age-adjusted incidence rate increased from 14.9 per 100,000 persons in 1975 to 23 per 100,000 persons in 2016. The joinpoint regression analysis similarly revealed two join points, as for the total population (Fig. 1). The incidence rate in males showed a rapidly increasing trend from 1975 to 1987 (APC = 3.6%, 95% CI = 3.0–4.2%, *P* < 0.0001), which slowed markedly from 1988 to 2003 (APC = 0.4%, 95% CI = 0.1–0.7%, *P* < 0.0001), before decreasing from 2004 to 2016 (APC = −0.9%, 95% CI= −1.3% to −0.6%, *P* < 0.0001).

Among female UCB patients, the age-adjusted incidence rate increased slightly from 3.8 per 100,000 persons in 1975 to 5.3 per 100,000 persons in 2016. Only one join point was identified (Fig. 1), with the incidence rate showing a slowly increasing trend from 1975 to 1996 (APC = 2.0%, 95% CI = 1.7–2.4%, *P* < 0.0001), followed by a slowing decreasing trend from 1997 to 2016 (APC = −0.8%, 95% CI = −1.2% to −0.5%, *P* < 0.0001).

### Characteristic of patients in predictive model

This study included 11,512 postsurgery UCB patients, with 8058 allocated to the training cohort and 3454 to the validation cohort. The median follow-up time was 37 months (range 1–119 months). Overall, 2607 (22.65.0%) patients died of postsurgery UCB during the follow-up. The basic characteristic of the patients are listed in Table 1. The total cohort was aged 70.35 ± 11.71 years, and included 8980 (78.01%) male patients. There were 10,240 (88.95%) white patients, 656 (5.70%) black patients, and 616 (5.35%) patients of other ethnic groups. The marital status was divided into 7209 (62.62%) married patients, 1471 (12.78%) unmarried or domestic partner or single patients, and 2832 (24.60%) separated or divorced or widowed patients. There were 11,138 (96.75%) insured and 374 (3.25%) uninsured patients. The primary tumor site in the largest proportion of patients was the anterior, posterior, and lateral walls (*n* = 4212, 36.61%), with overlapping lesions/location NOS in 5539 (48.12%) patients. Most of patients had an undifferentiated grade (*n* = 7082, 61.52%) or poorly differentiated grade (*n* = 2473, 21.48%), had a localized metastasis stage (*n* = 9,688, 84.16%), and were at AJCC stage I (*n* = 7461, 64.81%) or II (*n* = 2312, 20.08%).

**Table 1:** Baseline characteristic of the patients with urothelial cancer of the bladder

Factors	Training cohort (***n*** = 8058)	%	Validation cohort (***n*** = 3454)	%	Total (***n*** = 11512)	%	***P***
**Age**^1^	70.20 ± 11.61		70.68 ± 11.92		70.35 ± 11.71		0.04
**Sex**							0.07
Male	6322	78.46	2658	76.95	8980	78.01	
Female	1736	21.54	796	23.05	2532	21.99	
**Grade**							0.99
Well	309	3.83	134	3.88	443	3.85	
Moderately	1059	13.14	455	13.17	1514	13.15	
Poorly	1735	21.53	738	21.37	2473	21.48	
Undifferentiated	4955	61.49	2127	61.58	7082	61.52	
**Ethnic group**							0.97
White	7171	88.99	3069	88.85	10240	88.95	
Black	458	5.68	198	5.73	656	5.70	
Other	429	5.32	187	5.41	616	5.35	
**Marital status**							0.06
Married	5098	63.27	2111	61.12	7209	62.62	
Single	1025	12.72	446	12.91	1471	12.78	
SDW	1935	24.01	897	25.97	2832	24.60	
**Insurance**							0.41
Yes	7789	96.66	3349	96.96	11138	96.75	
No	269	3.34	105	3.04	374	3.25	
**Location**							0.19
Urachus/dome	303	3.76	140	4.05	443	3.85	
Trigone, neck, ureteric orifice	951	11.80	365	10.57	1316	11.43	
Wall	2920	36.24	1294	37.46	4214	36.61	
NOS/overlap	3884	48.20	1655	47.92	5539	48.12	
**Stage**							0.82
Localized	6782	84.16	2906	84.13	9688	84.16	
Regional	953	11.83	417	12.07	1370	11.90	
Distant	323	4.01	131	3.79	454	3.94	
**AJCC**							0.41
I	5214	64.71	2247	65.06	7461	64.81	
II	1627	20.19	685	19.83	2312	20.08	
III	499	6.19	236	6.83	735	6.38	
IV	718	8.91	286	8.28	1004	8.72	
**Size**							0.26
1-20 mm	1647	20.44	746	21.60	2393	20.79	
21-49 mm	3650	45.30	1516	43.89	5166	44.87	
≥ 50 mm	2761	34.26	1192	34.51	3953	34.34	
**Regional lymph nodes removed**							0.22
Yes	1574	19.53	641	18.56	2215	19.24	
No	6484	80.47	2813	81.44	9297	80.76	
**Chemotherapy**							0.45
Yes	2652	32.91	1162	33.64	3814	33.13	
No	5406	67.09	2292	66.36	7698	66.87	
**Radiation**							0.42
Yes	493	6.12	225	6.51	718	6.24	
No	7565	93.88	3229	93.49	10794	93.76	

*SDW* separated or divorced or widowed, *AJCC* American Joint Committee on Cancer

^1^The age was showed as mean ± standard deviation

In terms of treatment modalities, the regional lymph nodes were removed in 2215 (19.24%) patients, 3814 (33.13%) had received chemotherapy, and 718 (6.24%) had received radiation. There were no significant differences between the training and validation cohorts in sex, ethnic group, tumor size, marital status, insurance status, differentiate grade, metastasis stage, AJCC stage, tumor location, regional lymph nodes removal status, chemotherapy status, or radiation status (*P* > 0.05). The patients in the validation cohort were slightly older than those in the training cohort (*P* = 0.04). The log-rank test showed that the survival time did not differ significantly between the training and validation cohorts (*P* = 0.3).

### Independent prognostic factors and construction of the nomogram

**Fig. 2:**
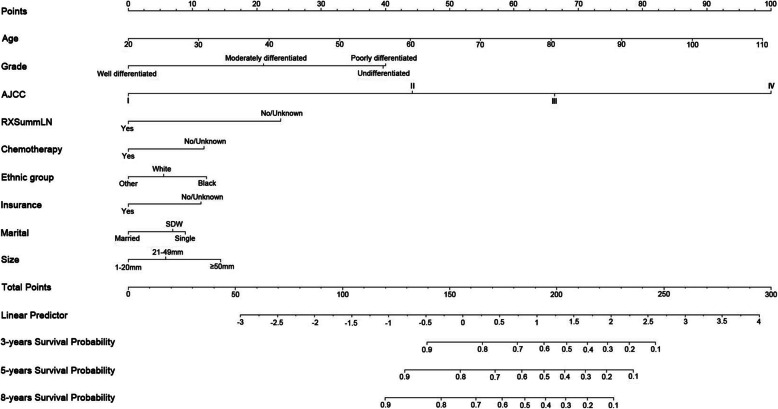
Nomogram for predicting 3-, 5-, and 8-year cancer-specific survival of postsurgery urothelial cancer of the bladder. When using the nomogram, each variable is assigned a score, and the scores for all of the variables are added to obtain the total score. A vertical line is then dropped down from the row showing the total scores to estimate the 3-, 5-, and 8-year survival rates. AJCC, American Joint Committee on Cancer; SDW, separated or divorced or widowed

Multivariable Cox regression with the backward stepwise selection method revealed that the statistically significant factors affecting postsurgery UCB survival in the training cohort were the age at diagnosis (hazard ratio [HR] = 1.03, 95% confidence interval [CI] = 1.03–1.04), black ethnic group (versus white: HR = 1.22, 95% CI = 1.01–1.47), moderately differentiated grade (versus well-differentiated grade: HR = 1.82, 95% CI = 1.11–2.99), poorly differentiated grade (versus well-differentiated grade: HR = 3.21, 95% CI = 2.00–5.15), undifferentiated grade (versus well-differentiated grade: HR = 3.13, 95% CI = 1.96–5.00), AJCC stage II (versus AJCC stage I: HR = 3.49, 95% CI = 3.08–3.96), AJCC stage III (versus AJCC stage I: HR = 5.92, 95% CI = 3.39–10.33), AJCC stage IV (versus AJCC stage I: HR = 12.97, 95% CI = 7.39–22.75), no regional lymph nodes removed (versus regional lymph nodes removed: HR = 1.82, 95% CI = 1.60–2.07), no chemotherapy (versus received chemotherapy: HR = 1.41, 95% CI = 1.27–1.57), no insurance (versus insured: HR = 1.40, 95% CI = 1.10–1.78), being single or unmarried or domestic partner (versus married, HR = 1.30, 95% CI = 1.13–1.50), being separated or divorced or widowed (versus married: HR = 1.22, 95% CI = 1.10–1.36), tumor size of 21–49 mm (versus 1–20 mm: HR = 1.18, 95% CI = 1.02–1.36), and tumor size of ≥ 50 mm (versus 1–20 mm: HR = 1.48, 95% CI = 1.28–1.71) (Table 2). These independent prognostic factors were used to construct a prognostic nomogram for predicting the 3-, 5-, and 8-year CSS of postsurgical patients with UCB (Fig. 2). The nomogram shows that the age at diagnosis and the AJCC stage were the strongest factors influencing the prognosis.

**Table 2:** The independent prognostic factors by multivariable Cox regression with the backward stepwise selection method in the training cohort

Factors	Hazard ratio	95% CI	***P***
**Age**	1.03	1.03-1.04	< 0.0001
**Grade**			
Well	1	**-**	**-**
Moderately	1.84	1.12-3.02	0.016
Poorly	3.19	1.99-5.12	< 0.0001
Undifferentiated	3.15	1.97-5.03	< 0.0001
**Ethnic group**			
White	1	**-**	**-**
Black	1.21	1.01-1.46	0.043
Other	0.85	0.69-1.06	0.146
**Marital status**			
Married	**1**	**-**	**-**
Single	1.29	1.12-1.49	0.0004
SDW^2^	1.22	1.1-1.36	0.0002
**Insurance**			
Yes	1	-	-
No	1.39	1.09-1.76	0.007
**AJCC**			
I	1	-	-
II	3.59	3.17-4.07	< 0.0001
III	6.8	5.74-8.07	< 0.0001
IV	18.04	15.6-20.86	< 0.0001
**Size**			
1-20 mm	1	-	-
21-49 mm	1.19	1.03-1.37	0.021
≥ 50 mm	1.52	1.31-1.75	< 0.0001
**Regional lymph nodes removed**			
Yes	1	-	-
No	1.99	1.76-2.24	< 0.0001
**Chemotherapy**			
Yes	1	-	-
No	1.41	1.27-1.56	< 0.0001

*SDW* separated or divorced or widowed, *AJCC* American Joint Committee on Cancer

### Validation of the nomogram

The C-index values of the nomogram were 0.820 and 0.823 in the training and verification cohorts, respectively, while those for the AJCC staging system were 0.773 and 0.786. Compared with the AJCC stage, the NRI values for 3, 5, and 8 years of follow-up were 0.34 (95% CI = 0.28–0.41), 0.37 (95% CI = 0.33–0.43), and 0.39 (95% CI = 0.34–0.44), respectively, in the training cohort, and 0.30 (95% CI = 0.20–0.40), 0.33 (95% CI = 0.21–0.41), and 0.32 (95% CI = 0.21–0.42) in the validation cohort; the corresponding IDI values were 0.057, 0.059, and 0.059 in the training cohort, and 0.042, 0.051, and 0.053 in the validation cohort (all *P* < 0.001). These performance indicators demonstrate that the nomogram showed better discrimination than the AJCC staging system.

**Fig. 3:**
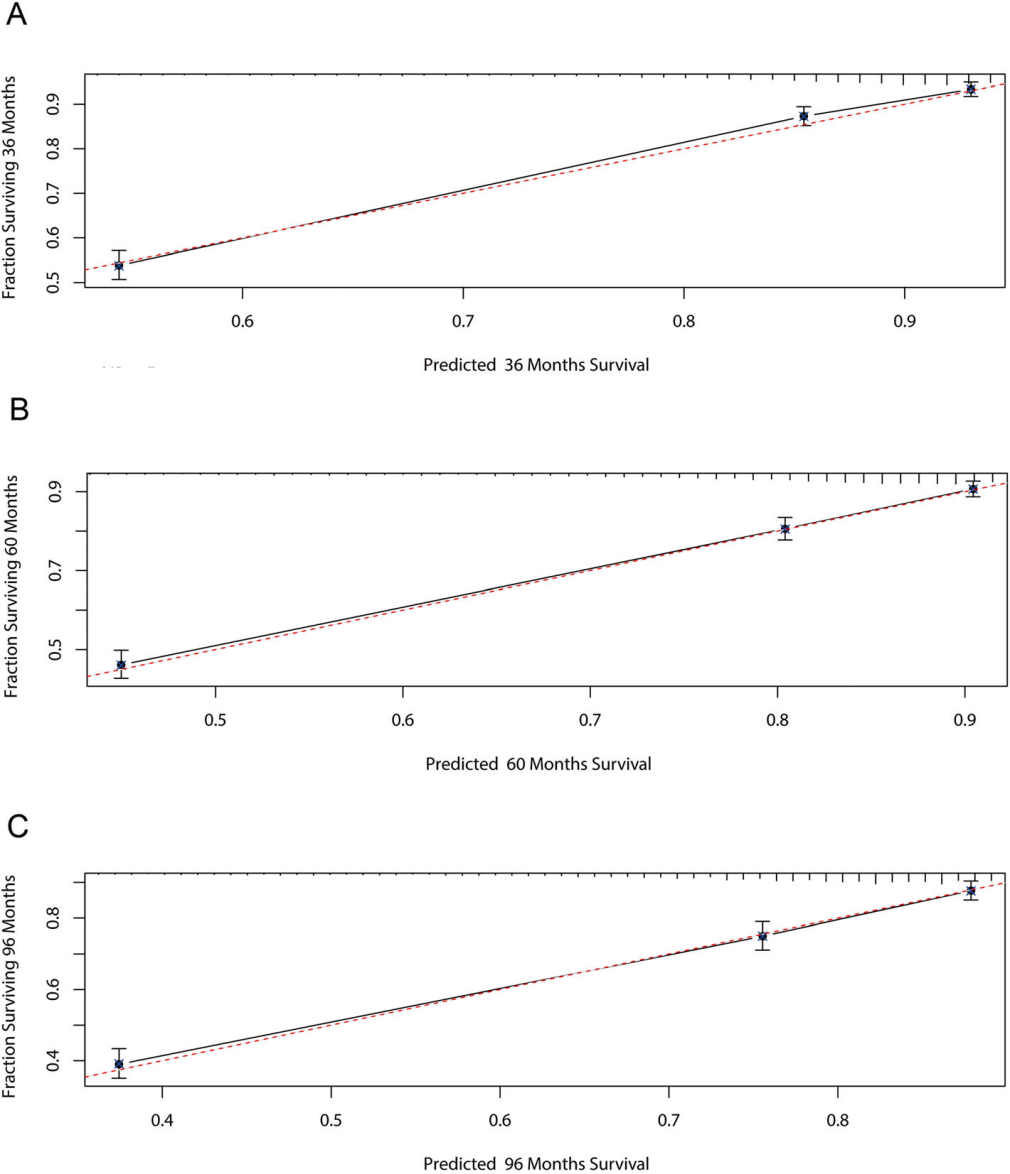
Calibration curves for 3- (**A**), 5- (**B**), and 8-year (**C**) cancer-specific survival of postsurgery urothelial cancer of the bladder in the validation cohort. X-axis, predicted survival probabilities by the nomogram. Y-axis, actual fraction surviving

**Fig. 4:**
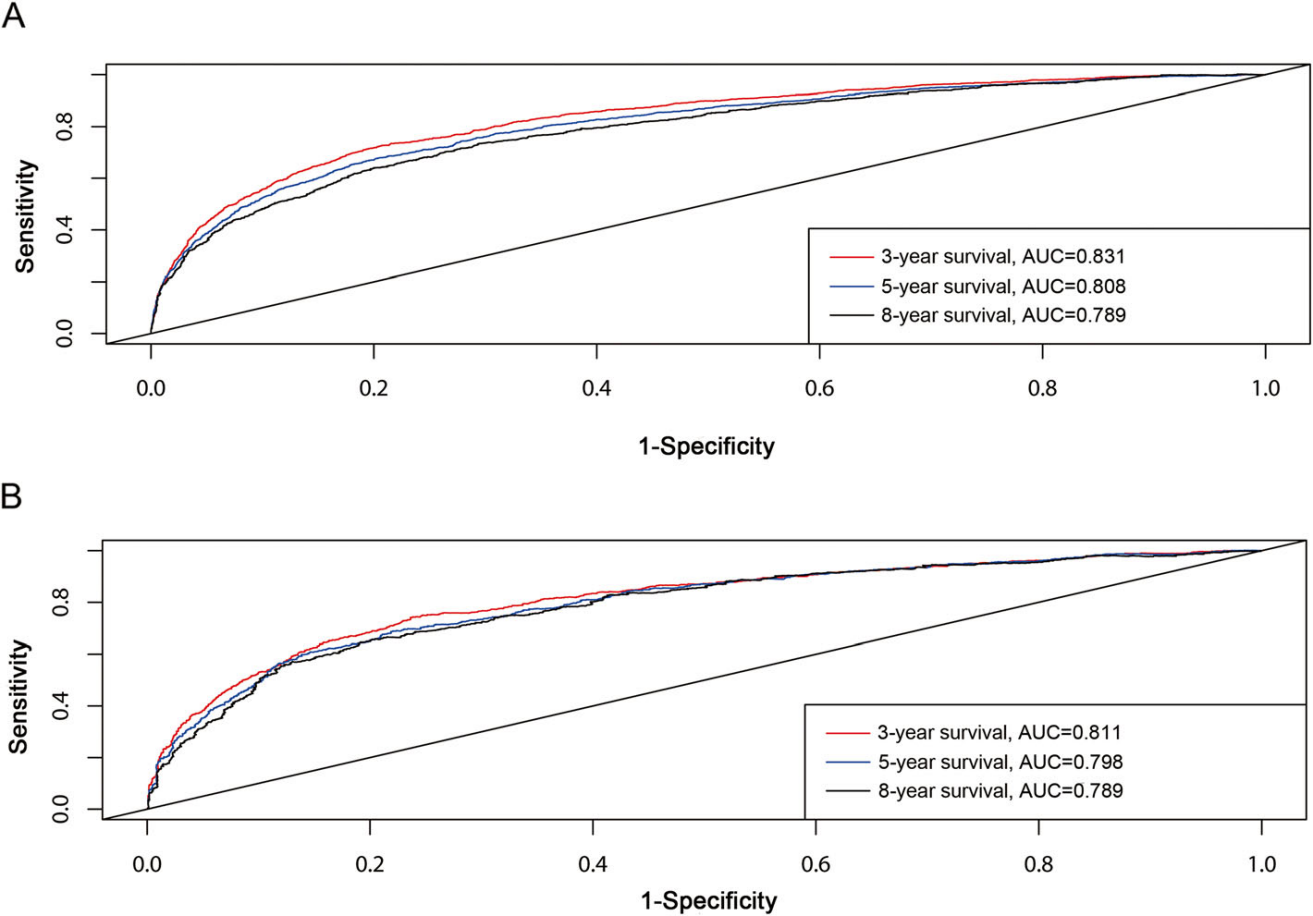
3-, 5-, and 8-year ROC curves in training (**A**) and validation cohorts (**B**) for validating nomogram model. ROC curve analyses were generated to test the performance evaluating of the new nomogram, by the areas under the ROC curves (AUC)

The calibration plots showed excellent consistency between the observed and nomogram-predicted probabilities in the training and validation cohorts (Fig. 3). The ROC curve of the predictive model showed good clinical effectiveness in both the training cohort (Fig. 4A), with areas under the ROC curve (AUCs) for 3, 5, and 8 years of follow-up of 0.831, 0.808, and 0.789, respectively, and the validation cohort (Fig. 4B), with corresponding AUCs of 0.811, 0.798, and 0.789.

## Discussion

This study analyzed incidence trends in order to establish a survival predictive model for postsurgery UCB based on data in the SEER database. From 1975 to 2016, the overall incidence rate showed an upward trend, despite a slight decrease from the beginning of the twenty-first century. Although management methods for UCB have evolved over the past 30 years, there has been no model for predicting the individualized CSS for UCB. The nomogram established in this study provides very accurate individualized prognostic predictions for postsurgery UCB patients, with good distinguishability and calibration.

The overall upward trend in the incidence of UCB over the past 40 years is consistent with the results of many studies, although the types of pathologies investigated have varied [18–22]. This increase is mostly attributable to progress in the development of diagnostic tools, especially in ultrasonography, computed tomography, and magnetic resonance imaging [23]. Another possible reason is the global trend of population aging, since this cancer is more common in the elderly, while the joinpoint regression also found that the incidence of UCB was not always rising, but had experienced a process of rapid rise, slow rise and then decline in men and the general population. We speculate the downward trend may be related to the control of tobacco consumption. Tobacco smoking is the main factor underlying the incidence of bladder cancer [24]. A report from the Centers for Disease Control and Prevention showed that the smoking rate has decreased markedly in American adults over the past few decades, from 42.4% in 1965 to 16.8% in 2014 [18]. It should be noted that there was long latency between tobacco exposition and bladder cancer diagnosis [25]. So the downward trend only began to appear around 2000. Another issue is that the incidence of female UCB had declined in earlier years. We suspect that the possible explanation is that women had a lower bladder cancer incidence because of potential biologic factors, and the decrease in tobacco consumption exerted a more significant impact on them.

Our study found that the prognosis is worse for postsurgery UCB patients who are single, separated, divorced, or widowed than it is for married patients. We speculate that this could be due to the mental status of UCB patients affecting their survival. It has been shown that single patients with bladder cancer are more likely to have a posttreatment psychiatric diagnosis than are married patients, and that the prognosis of bladder cancer is worse in patients with a psychiatric diagnosis [26]. Other analyses of the prognosis of bladder cancer using data from the SEER database have also found that the marital status can affect the prognosis of the disease [27, 28].

We further found that the prognosis is worse in patients without insurance than in those receiving medical insurance/medical assistance. This is somewhat consistent with the findings of Sung et al. [29] based on California Cancer Registry data that the survival time for bladder cancer is worse for not-insured patients and those with an unknown insurance status than it is for those with managed care, although there was no significant difference in the CSS. That study also found that among all insurance categories, the prognosis was worst for Medicaid insurance in the USA. We speculate that the main reason is that Medicaid is aimed at low-income people, who are less likely to receive treatment within 12 weeks of a diagnosis [30]. Sung et al. [29] also found that Medicaid patients had more advanced-stage, higher-grade tumors compared with patients covered by Medicare or managed care, and so their prognosis may be worse. This has been confirmed in other previous research [31]. In our study, we did not subdivide the patients into different types of insurance, instead only dividing them into insured and uninsured/unknown, which may be the main reason for the difference in the research results. Regardless, the type of and accessibility to medical insurance may affect the survival rate of bladder cancer, possibly due to differences in basic living conditions (e.g., income and living environment), disease prevention, and the treatment of people covered by different types of medical insurance.

Other independent prognostic factors for postsurgery UCB identified in this study were the age at diagnosis, black ethnic group, lower differentiation grade, lower AJCC stage, no regional lymph nodes removed, not receiving chemotherapy, and larger tumor, which is traditional prognostic factors for bladder cancer that have been reported previously [32–34]. Based on these factors and the aforementioned marital status and insurance status, we established a nomogram for the individualized prognosis of postsurgery UCB, and found that the AJCC stage and the age had the greatest impact on individualized prognoses. This was not surprising. The AJCC stage itself reflected the severity of the tumor to a large extent. On the other hand, the elderly patients usually suffered from reduced physiological function, coupling with other underlying diseases, resulting in that perioperative mortality and postoperative complications had increased significantly. Additionally, the risk of recurrence increased with age, and the prognosis of older patients was poor [35]. However, the contribution of other variables to the model cannot be ignored. We calculated the NRI and IDI of established model using “Age + AJCC stage” as the control model and found the NRI values for 3, 5, and 8 years of follow-up were 0.23, 0.2, and 0.17, respectively, in the training cohort, and 0.19, 0.12, and 0.12 in the validation cohort; the corresponding IDI values were 0.03, 0.03, and 0.03 in the training cohort, and 0.02, 0.02, and 0.03 in the validation cohort (all *P* < 0.001). These indicated that variables other than AJCC and age also exerted a positive contribution to the prediction of prognosis.

The nomogram developed in this study is the first one reported for postsurgery UCB. Zhang et al. [36] established a nomogram for the individualized prognosis of bladder cancer based on data in the SEER database. The variables in that model include the age at diagnosis, ethnic group, sex, and TNM stage. That model also indicated that age and the T stage have the greatest impact on the prognosis, which is essentially consistent with our model; the main differences are that we used AJCC staging, which is also based on the TNM stage, and we targeted postsurgery UCB. Our nomogram might be superior since we take into account the clinical treatment received by the patients and a broader range of demographic information. In addition, the nomogram that we have established exhibits good discrimination, calibration, and clinical effectiveness, and a better prognostic ability for postsurgery UCB than the currently used AJCC staging system. This easy-to-use nomogram can help doctors to estimate the likelihood that a patient will survive at a certain point in time.

Several limitations of this study should be considered. Firstly, the data used in the validation cohort also came from the SEER database, and so the nomogram still needs to be validated using data from another database or using clinical prospective data. Secondly, some important clinical factors were not collected, such as the smoking status after diagnosis, parameters of social status (e.g., socioeconomic status or level of education), condition of the underlying disease, comorbidities, and biochemical indicators such as the C-reactive protein level. The data available are also subject to the limitations of the SEER database. Finally, for patients with bladder cancer to have a good prognosis, preventing relapse is also an important indicator for the clinical treatment of the disease [37, 38], but we did not analyze the risk of recurrence in patients.

## Conclusions

In conclusion, this study has revealed the incidence trends of UCB and constructed a nomogram for predicting the long-term survival of individual postsurgery UCB patients based on a population cohort. The nomogram showed good predictive performance, and may serve as an effective and convenient evaluation tool for helping surgeons to perform personalized survival predictions and mortality risk identification in postsurgery UCB patients.

## Data Availability

The data that support the findings of this study are available on request from the corresponding author.

## Abbreviations

UCB: Urothelial cancer of the bladder
AJCC: American Joint Committee on Cancer
CSS: Cancer-specific survival
NRI: Net reclassification index
IDI: Integrated discrimination improvement
APC: Annual percentage change
ROC: Receiver operating characteristics
AUC: Areas under the ROC curve
HR: Hazard ratio
CI: Confidence interval
